# Death Pronouncement: Preparing Incoming Residents for Duties When Life Ends

**DOI:** 10.7759/cureus.25275

**Published:** 2022-05-24

**Authors:** Zaid Kaloti, Renieh Nabaty, Abubekr Mohamed, Sarvani Surapaneni, Anthony Gaynier, Diane L Levine

**Affiliations:** 1 Internal Medicine, Wayne State University Detroit Medical Center, Detroit, USA; 2 Medicine, Wayne State University School of Medicine, Detroit, USA; 3 Medical Education, Wayne State University School of Medicine, Detroit, USA

**Keywords:** end-of-life and hospice care, end-of-life care, death pronouncement, formal education death pronouncement, transition to residency curriculum, duties when life ends, medical education

## Abstract

Introduction

Undergraduate Medical Education (UME) prepares future residents for many aspects of medical practice, but it is rarely all-inclusive. Death pronouncement (DP), a highly important aspect of clinical training for residents, seems to be inadequately addressed and taught in undergraduate institutions. Studies have indicated that most first-year residents received minimal DP training and felt unprepared for this duty. Despite being a challenging situation, a formal teaching course is not universally taught, with most institutions merely delivering point-of-care DP instruction to medical trainees provided by supervising faculty, senior residents, and nurses. Our primary objective was to provide formal education in Duties When Life Ends (DWLE), with the goal of enhancing familiarity, knowledge, and confidence in addressing the circumstances surrounding death for graduating medical students transitioning to residency.

Methods

As a part of a Transition to Residency (TTR) course for students entering nonsurgical specialties, we developed a curriculum to provide formal education to fourth-year medical students in DWLE that included a two-hour didactic session delivered virtually, followed by an in-person simulation session. The didactic session covered the history, processes of DP, death physical examination, identification of medical examiner (ME) case, education on how to deliver death news to family, information about autopsies and organ donation, distinction between the cause and mechanism of death, and documentation of death notes and certificates, as well as provider self-reflection and appropriate coping mechanisms for patient death.

In the 45-minute simulation, students were divided into small groups and given a case summary. During the first half, they performed a physical examination and a verbal pronouncement on cadavers, followed by an interactive small group session where students reviewed the case and worked to identify the cause of death, determine if the death was a medical examiner’s case, deliver death news to the family, and complete a death progress note and certificate. Pre- and post-session questionnaires were administered, assessing three components: process familiarity, knowledge, and confidence. Finally, participants assessed course usefulness and had a free response opportunity for comments and feedback.

Results

Overall, 198 students participated in all sessions, with 182 completing both pre- and post-session questionnaires. Pre-survey revealed that 70% of participants reported witnessing DP previously, with only 20% being familiar with the process of DP and 6% with documentation. Following the intervention, a comparison of the pre- and post-course questionnaires assessing process familiarity, knowledge, and confidence using a five-point Likert scale demonstrated statistically significant improvement in the mean scores in all three domains, with reported course usefulness of 96%.

Conclusion

A DWLE curriculum, as a part of the TTR course, was effective in improving self-reported familiarity, knowledge, and confidence regarding physician duties associated with patient death. The curriculum was well received by students. The incorporation of DWLE curriculum into TTR courses allows for vital preparation and education in the duties related to patient death. This may make a stressful process somewhat less stressful and may aid future physicians in developing competence in conducting these final physician duties.

## Introduction

Undergraduate Medical Education (UME) prepares medical students for many aspects of medical practice as future residents. However, it is rarely all-encompassing. Basic sciences such as anatomy, physiology, pathology, and pharmacology are heavily covered, but topics such as cost-effective care, medical-legal issues, and end-of-life care receive less attention [1]. A study by Chen et al. found that about 54% of residents believed that medical school had adequately prepared them for residency; however, only 42% of residents agreed that their UME had prepared them well for providing end-of-care needs and basic palliative care [1].

End-of-care curricula focus on caring for patients at the end of life; however, duties to patients do not end when life ends. One of these duties includes death pronouncement (DP). One study showed that less than 30% of first-year residents had any form of DP training in medical school, with the majority needing supervision or assistance with the process [2]. Despite DP being a challenging situation, a formal course or curriculum addressing this may not be provided in most undergraduate medical institutions; rather, medical students and, oftentimes, residents receive informal DP education during their medical clerkships or residency rotations [2]. This informal training in DP is often completed by supervising faculty, senior residents, and nurses on a point-of-care basis [3-5]. In a survey of 175 residents, only half felt very comfortable performing DP tasks, with most reporting that they had learned to pronounce death from their senior resident and that a minority had no DP training [4]. Another survey found that only 36% of participating attending physicians reported formal training in DP during their medical training [6]. The delivery of formal training sessions and workshops has been limited, with only some postgraduate residency programs incorporating and researching this into their training [3-7]. For instance, one workshop conducted for Family Medicine residents incorporated six Accreditation Council on Graduate Medical Education (ACGME) competencies into the training, reflection of DP, and patient family discussions, allowing residents to become more prepared for this daunting task [3].

Medical students have little exposure to death and dying patients during clinical rotations and even less confidence in their skill set for caring for dying patients and the vital discussions with surviving families [8]. With many undergraduate medical institutions offering Transition to Residency (TTR) courses, it is unknown how many institutions are incorporating DP training into their curriculum. However, multiple studies have found that resident trainees are underprepared for completing tasks associated with DP, leaving the burden of DP training to postgraduate residency programs [3-7]. Formal training in the senior year of medical school may facilitate the development of more prepared and competent future residents.

In this paper, we describe a structured curriculum surrounding Duties When Life Ends (DWLE) in which medical students in their final year were provided a virtual didactic session and an in-person simulation on DP as a part of the Transition to Residency course. Our primary objective was to provide formal education in DWLE, with the goal of enhancing familiarity, knowledge, and confidence in addressing the circumstances surrounding death for graduating medical students transitioning to residency.

## Materials and methods

Through an informal needs assessment at a large public urban medical school, in Detroit, Michigan, we identified a gap in the training of fourth-year medical students regarding physician duties and responsibilities surrounding the death of a patient. A process map of these duties was created and reviewed with residents in the Internal Medicine residency training program affiliated with our School of Medicine (SOM). Six distinct duties were identified: DP, identification of a medical examiner’s (ME) case, delivery of death news, request of an autopsy, documentation of death in the medical record, and completion of the death certificate. After reviewing the literature, we developed a curriculum to provide formal education addressing these six areas as part of our institution’s Transition to Residency (TTR) course for nonsurgical specialties. The curriculum included a two-hour didactic session delivered virtually, followed by a 45-minute, two-part, in-person simulation session.

Virtual component

The virtual didactic session covered components of DWLE, including the history, processes of DP, death physical examination, identification of medical examiner case, education on how to deliver death news to the family, information about autopsies and organ donation, documentation of the death note, and education about the distinction between the cause and mechanism of death as related to the completion of the death certificate. The session included a discussion about provider self-reflection and appropriate coping mechanisms for patient death. The two-hour didactic session included two practice cases to allow students to complete death certificates. At the end of the session, the students received a handout summarizing the information provided.

In-person simulation

The in-person simulation was subdivided into two sessions lasting a total of 45 minutes. The learning objective for the first 15-minute session was to demonstrate a death examination and verbal DP. The students were divided into groups of 5-6, given a case summary, and brought to the cadaver laboratory. Each student performed a physical examination and a verbal pronouncement on lightly preserved cadavers. The students were directly observed by an academic clinic faculty member and provided immediate feedback regarding DP and the professionalism associated with this task, including how they entered and exited the room. This was followed by a 30-minute interactive small group session.

During the interactive small group session, case summaries were reviewed. Roles and responsibilities were divided among students, including reviewing the case summary to identify the cause of death, determining if the death was a medical examiner’s (ME) case, conducting a simulated call to the ME, and delivering death news to a family during role play. Death progress notes and certificates were completed as a group. Each group was assigned a faculty member, who played the role of the ME and later the grieving family member. The faculty reviewed documentation with the group and provided post-session debriefing using a standardized script.

Questionnaire and analysis

The participants completed a pre-curriculum questionnaire prior to the virtual didactic session and a post-curriculum questionnaire following the in-person session. There was a total of 23 questions assessing the components of the curriculum: process familiarity, self-reported knowledge, and confidence, using a five-point Likert scale (Table 1). The post-curriculum questionnaire was identical to the pre-curriculum questionnaire. It also included one additional question regarding course usefulness and a free-response opportunity for comments and feedback. The means for each question were calculated and compared. Significance was defined at P<0.05. The study was reviewed by the IRB and did not require formal approval because the data collected was confidential, voluntary, and used to improve and for the development of educational programs for the students at our institution.

**Table 1:** Pre- and Post-curriculum Questionnaire

Questionnaire	
Familiarity: How familiar are you with the following?	1. Pronouncement of a dead patient
	2. Completion of a death certificate
Knowledge: Rate your knowledge on the following.	1. Components of a physician in documenting a death pronouncement
	2. Manner in which one should inform the patient’s family about the death of the patient
	3. Dos and don’ts of a telephone death notification
	4. Circumstances necessitating calling a medical examiner
	5. Dispelling myths about organ donation
	6. Resources available to patient’s survivors when informing about a death
	7. Resources available to you
	8. Communication strategies in breaking bad news
	9. Differences between the cause of death and mechanism of death
	10. Components of a death note and certificate in Michigan, USA
Confidence: How confident are you in the following?	1. Documenting a death pronouncement
	2. Determining families’ understanding of the patient’s condition before informing of death
	3. Informing survivors about the death of a loved one
	4. Dealing with emotions from survivors
	5. Requesting an autopsy
	6. Requesting organ donation
	7. Identifying resources to assist survivors during a death notification
	8. Completing death note and certificate
	9. Identifying the principal cause of death
	10. Identifying the underlying causes of death
	11. Identifying the mechanism of death
Post-session	How useful was this course?

## Results

**Figure 1:**
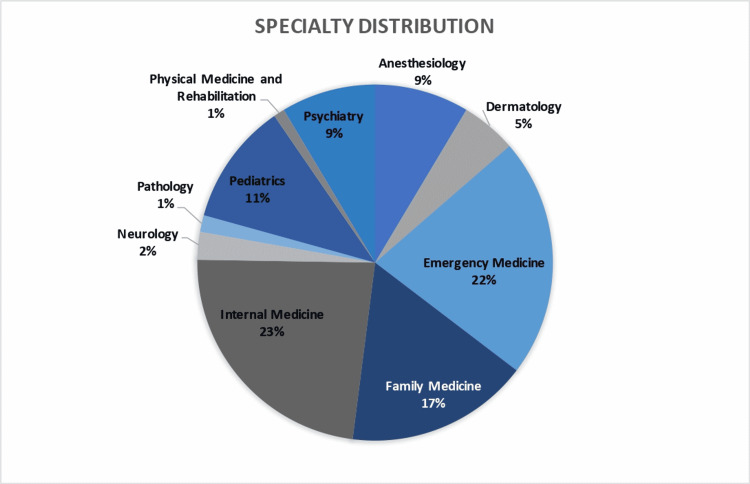
Specialty Distribution of Students Who Participated in the Nonsurgical Transition to Residency Course (N=198)

A total of 198 senior medical students participated in the DWLE curriculum as part of a larger TTR course, which was delivered in March and April of 2021. The distribution of future specialties based on match results of students participating in the course is seen in Figure 1.

Of the 198 participating students, 182 students (n=182, 92%) completed both the pre- and post-curriculum questionnaire, with a completion rate of 92%. Pre-questionnaire revealed that 70% of the participants reported witnessing DP previously, with only 20% being familiar with the process of DP and 6% with documentation. Overall, there was a statistically significant improvement between the pre- and post-curriculum average mean score in familiarity, self-reported knowledge, and confidence (Table 2).

**Table 2:** Overall Improvement Between Pre- and Post-curriculum in Three Categories

Category	Mean Pre-curriculum	Mean Post-curriculum	P<0.001
Familiarity	2.21	4.10	
Knowledge	2.03	3.95	
Confidence	2.61	4.15	

Process familiarity

**Figure 2:**
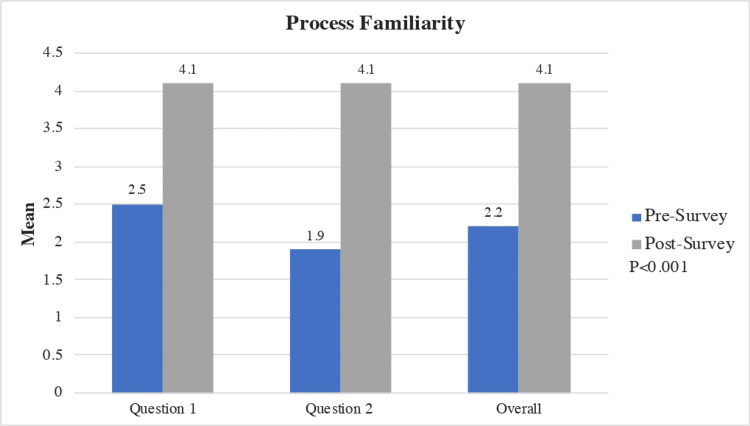
Mean Improvement in Process Familiarity, Pre- and Post-curriculum Questionnaire, and Overall Process Familiarity Improvement

In this category, there was a statistically significant increase in each question related to familiarity with DWLE and completion of a death certificate between the pre- and post-curriculum questionnaires. Following the intervention, statistically significant improvement in process familiarity and documentation was seen at 90% and 92%, respectively (Figure 2).

Knowledge

**Figure 3:**
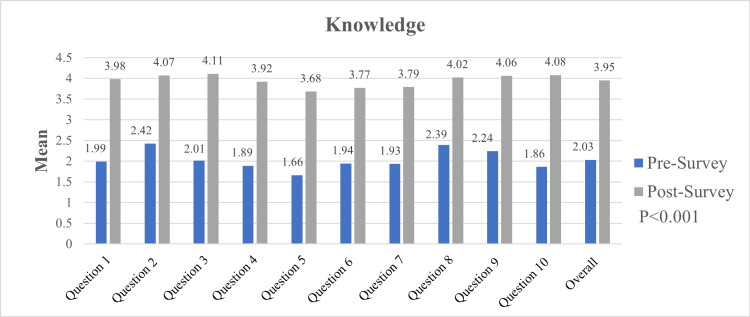
Mean Improvement in Self-Reported Knowledge, Pre- and Post-curriculum Questionnaire, and Overall Knowledge Improvement

In regard to self-reported knowledge, the overall mean had a statistically significant improvement from 2.0 to 3.9. The most substantial difference was seen in question 3, corresponding with the dos and don’ts of a telephone death notification (mean improvement from 2.01 to 4.11), and question 10, corresponding with the components of a death note and certificate (mean improvement from 1.86 to 4.08) (Figure 3).

Confidence

**Figure 4:**
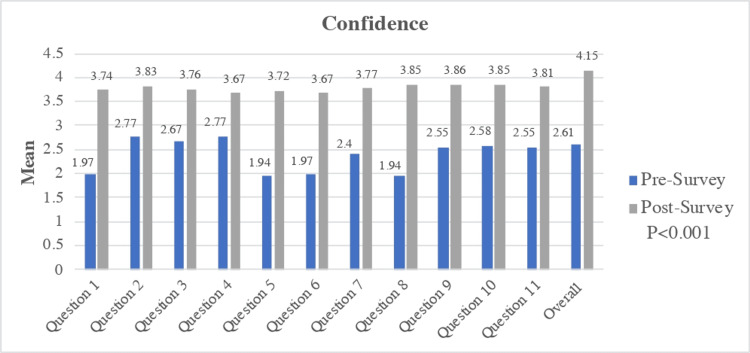
Mean Improvement in Confidence, Pre- and Post-curriculum Survey, and Overall Confidence Improvement

There was a similar statistically significant improvement from pre- to post-session in confidence, with the mean confidence level increasing from 2.61 to 4.15. The most substantial improvement was seen in the confidence question 5, corresponding with requesting an autopsy (mean improvement from 1.94 to 3.72), and question 8, corresponding with completing a death note and certificate (mean improvement from 1.94 to 3.85) (Figure 4).

**Figure 5:**
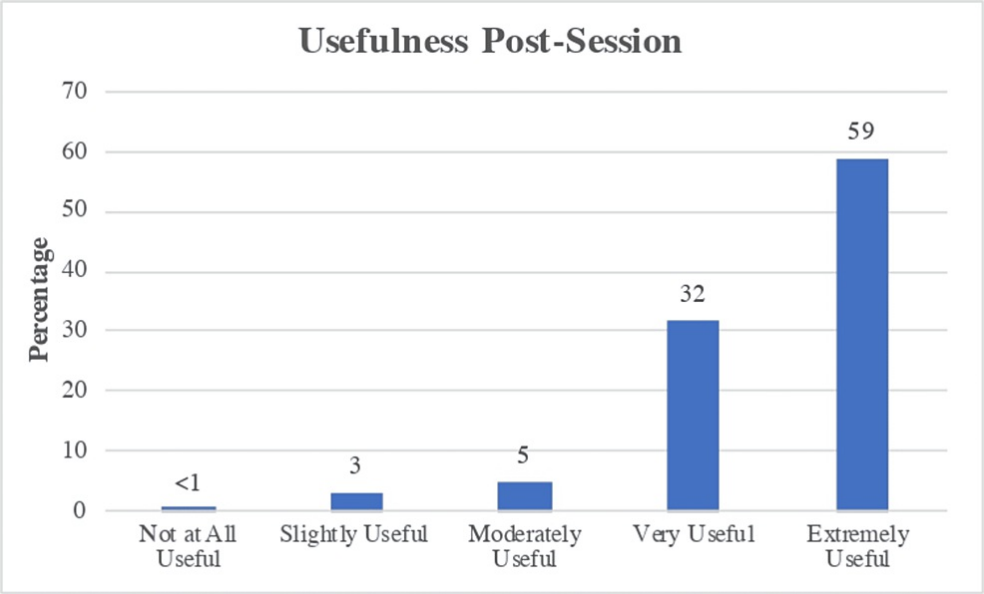
Post-curriculum Questionnaire Results Assessing Course Usefulness

The last question on the post-curriculum questionnaire addressed course usefulness on a five-point scale from “Not at All Useful” to “Extremely Useful.” The overall course usefulness (from “Moderately Useful” to “Extremely Useful”) was reported to be at 96% (Figure 5).

## Discussion

Our study showed that, despite most senior medical students reporting witnessing or attending some DP during their clinicals, the majority of the students lacked familiarity with the process of DP and with the DWLE. The majority reported “very poor to poor” knowledge and confidence in this important subject. We demonstrated that a two-hour virtual session followed by a 45-minute simulation addressing the roles and responsibilities surrounding death resulted in increased familiarity, knowledge, and confidence in DWLE.

Hawkins et al. found that medical students have little exposure to death and dying patients during clinical rotations, contributing to less confidence in their skill set in caring for dying patients and the vital discussions with surviving families [8]. In contrast, the majority of our students witnessed patients dying, death, and DP. However, despite exposure, only a minority were familiar with the DWLE, and most lacked familiarity with the entire process.

Exposure to death and the process of DP is oftentimes insufficient for graduating medical students to perform DWLE once they become residents, leading to greater dependence on nonstandardized informal experience led by supervising faculty, residents, and nursing staff [3-5]. In a large survey of US medical graduates, Chen et al. found that, although residents felt “well prepared” by medical school training in basic sciences and clinical proficiencies, a significant portion of the surveyed residents felt unprepared to cope with personal and professional matters pertaining to end-of-life care [1]. This emphasizes the importance of teaching DWLE education at all levels of medical training including students and residents [9,10].

There is a paucity of literature on teaching DWLE with most focusing on DP [11]. Additionally, although many schools deliver education around end-of-life care, there is less published about delivering death news [11]. Physician-patient-family communication and physician behavior during a DP is an important component of DWLE. This communication leaves a lasting impression on families, which has the potential to either positively or negatively impact the family during the bereavement process [11-13].

The Coalition for Physician Accountability, a group of national organizations responsible for the accreditation, assessment, licensure, and certification of physicians from medical school through practice, recently outlined recommendations for Comprehensive Improvement of the Undergraduate-Graduate Medical Education Transition [14]. Recommendation number 28 states, “Specialty-specific, just-in-time training must be provided to all incoming first-year residents, to support the transition from the role of student to a physician ready to explain, increased responsibility for patient care” (Coalition, 2021, p. 24). In their narrative description, they go to explain, “residents reported greater preparedness for residency if they participated in a medical school “boot camp,” and participation in longer residency preparedness courses was associated with high perceived preparedness for residency” (Coalition, 2021, p. 24). “Boot camps” also known as Transition to Residency courses are increasingly being offered to medical students, with the number of institutions offering specialty-specific TTR courses more than doubling from 2014-2015 to 2020-2021 [15].

Preparing students to become physicians ready to deliver care under indirect supervision is the responsibility of medical schools. We believe that these duties include preparing newly minted physicians for DWLE. A Transition to Residency course, as part of undergraduate education, is an ideal time to provide just-in-time care to students who have the clinical experience and maturity to appreciate the importance of this curriculum.

Limitations

Our study has limitations. Our curriculum was conducted at a single medical school involving students going into nonsurgical specialties and may not be generalizable to other medical schools or students going into other specialties. Additionally, this study is based on the self-perceived improvement of familiarity, knowledge, and confidence in medical students following the curriculum. However, there was no form of standardized evaluation in place to measure objective improvements in DP knowledge and skills. The use of role play as a means of learning the DWLE curriculum may serve as another limitation. There is evidence suggesting that learning via role play in the presence of peers and artificial scenarios may limit the learning experience, ultimately, being reflected in the lack of confidence of young doctors who trained in such methods [8]. Additionally, role play may not be translatable to the actual practice of DP and other DWLE. Finally, the in-person simulation took place in a cadaver laboratory. This may serve as a limitation in the application of this simulation to real-life clinical practice.

This study has shown that medical students, at baseline, may not have adequate understanding on the DWLE, which negatively impacts the performance of this important clinical duty as a resident physician. The creation of a formal curriculum that addresses this duty as a part of a Transition to Residency course allows for substantial improvement in the familiarity, knowledge, and confidence of graduating medical students in the DWLE.

## Conclusions

A DWLE curriculum, as a part of the Transition to Residency course, was effective in improving self-reported familiarity, knowledge, and confidence regarding physician duties associated with patient death. The curriculum was well received by students. The incorporation of DWLE curriculum into a Transition to Residency course allows for vital preparation and education in the duties related to patient death. This may make a stressful process somewhat less stressful and may aid future physicians in developing competence in conducting these final physician duties.
